# Circ-ATAD1 is overexpressed in osteosarcoma (OS) and suppresses the maturation of miR-154-5p to increase cell invasion and migration

**DOI:** 10.1186/s13018-021-02809-4

**Published:** 2021-12-02

**Authors:** Jihui Zhou, Li Xu, Peng Yang, Shibang Lin, Haizhou Huang

**Affiliations:** 1 Department of Traumatic Orthopedics, Maoming People’s Hospital, No. 101 Weimin Road, Maoming City, 525000 Guangdong Province People’s Republic of China; 2 The 9th Recuperation Department of Qingdao Special Servicemen Recuperation Center of PLA NAVY, Qingdao City, 266071 Shandong Province People’s Republic of China; 3 Department of Hand and Foot Surgery, The Eighth People’s Hospital of Qingdao, Qingdao City, 266100 Shandong Province People’s Republic of China

**Keywords:** Osteosarcoma, Circ-ATAD1, miR-154-5p, Maturation, Migration, Invasion

## Abstract

**Background:**

Circ-ATAD1 plays an oncogenic role in gastric cancer. However, its roles in other cancers are unclear. We aimed to analyze the role of circ-ATAD1 in osteosarcoma (OS).

**Methods:**

The expression levels of circ-ATAD1, mature miR-154-5p, and premature miR-154-5p in paired OS and non-tumor tissues from 56 OS patients were determined using RT-qPCR. Nuclear fractionation assay was performed to analyze the subcellular location of circ-ATAD1. The interaction between circ-ATAD1 and premature miR-154-5p was analyzed using RNA pull-down assay. The role of circ-ATAD1 in regulating miR-154-5p maturation was analyzed using RT-qPCR in cells with overexpression. Transwell assays were performed to analyze the roles of circ-ATAD1 and miR-154-5p in regulating OS cell invasion and migration.

**Results:**

Circ-ATAD1 was overexpressed in OS compared to non-tumor tissues and was detected in the nuclei of OS cells. Mature miR-154-5p, but not premature miR-154-5p, was downregulated in OS tissues compared to non-tumor tissues and was inversely correlated with circ-ATAD1. In OS cells, circ-ATAD1 overexpression decreased the expression of mature miR-154-5p, but not premature miR-154-5p. Transwell assay analysis showed that circ-ATAD1 overexpression increased cell invasion and migration, and mature miR-154-5p overexpression suppressed these cell behaviors. In addition, circ-ATAD1 overexpression reduced the effects of mature miR-154-5p overexpression on cell behaviors.

**Conclusions:**

Circ-ATAD1 is overexpressed in OS and suppresses miR-154-5p maturation to increase cell invasion and migration.

**Supplementary Information:**

The online version contains supplementary material available at 10.1186/s13018-021-02809-4.

## Introduction

Osteosarcoma (OS), also refers to osteogenic sarcoma, is the most common type of bone malignancies [1]. OS mainly affects young adults, teenagers, and children [2, 3]. It is estimated that OS affects about 4.4 out of 1 million people younger than 25 years, with a lower incidence in whites than in blacks [2, 3]. OS patients diagnosed at early stages can usually be cured after combined therapies, including surgical resection, radiotherapy, and chemotherapy [4]. However, distant tumor metastasis is frequently observed by the time of initial diagnosis of OS [5]. In addition, postoperative recurrence is also common [6], leading to poor survival. Therefore, treatment of OS still requires the development of novel approaches.

With the advantages of fewer adverse effects and high efficiency, molecular targeted therapy, which can be applied to regulate cancer-related gene expression, has shown potentials in the treatment of cancers, including OS [7–10]. For instance, insulin-like growth factor-2 receptor has been proven to be a potential target to treat OS [8]. However, more molecular targets are needed to further improve OS-targeted therapy. Circular RNAs (circRNAs) have no or limited coding capacity, but they affect cancer development mainly by regulating the expression of protein-coding genes [11, 12], suggesting that circRNAs are potential targets for cancer therapy. Circ-ATAD1 has been reported to play an oncogenic role in gastric cancer [13]. Our preliminary microarray data have shown that circ-ATAD1 expression is altered in OS and inversely correlated with miR-154-5p to which suppresses OS [14]. Based on our knowledge, the crosstalk between circ-ATAD1 and miR-154-5p has not been reported previously. Therefore, we analyzed the interaction between circ-ATAD1 and miR-154-5p in OS.

## Materials and methods

### Paired tissue samples

No circ-ATAD1 expression data were found in public datasets, such as TCGA. Therefore, to analyze the differential expression of circ-ATAD1, this study included 56 OS patients who were willing to donate tissue samples. All patients were enrolled at Maoming People's Hospital after the Ethics Committee of this hospital approved this study. The 56 patients included 30 males and 26 females, with a mean age of 19.9 ± 4.7 years. All patients and their patients signed informed consent. Surgical resection was performed on these patients. Tumors were dissected by experienced histopathologists to separate tumor tissues (OS) and normal bone tissues at the edge of tumors (non-tumor). Based on pathological analysis, the 56 patients included 38 cases at AJCC stage I or II and 18 cases at stage III or IV. Patients complicated with other severe clinical disorders or subjected to treatments prior to admission were excluded.

### OS cells and transfections

Two OS cell lines, Hs 3.T and MG-63, and normal bone cell line Hfob1.19 (ATCC, USA) were used in this study. Cell culture was carried out following the manufacturer’s instructions. Circ-ATAD1 and miR-154-5p overexpression and circ-ATAD1 silencing were reached in Hs 3.T and MG-63 cells by transfecting pcDNA3.1-circ-ATAD1 expression vector, mimic of miR-154-5p, or circ-ATAD1 siRNA (RiboBio) using Neon Transfection System (Thermo Fisher Scientific). All operations were performed following the manufacturer’s instructions. Overexpression was confirmed by RT-qPCR every 24 h until 72 h or 96 h. Empty vector or miRNA mimic were included as the negative controls. Un-transfected cells were used as controls.

### RNA sample preparation

Total RNAs were extracted from cells from each transfection group and paired tissues from each patient using Ribozol reagent (Invitrogen) and treated with DNase I (Sangon) to remove genomic DNAs until an OD 260/280 ratio close to 2.0 was reached. RNA integrity was analyzed by separating RNAs on 5% urea-PAGE gels.

### RT-qPCRs

To determine circ-ATAD1 and premature miR-154-5p expression in cells and tissues, RNA samples were first reverse transcribed into cDNA samples and analyzed using qPCRs with 18S rRNA as the internal control. Mature miR-154-5p expression in RNA samples was analyzed using All-in-One™ miRNA qRT-PCR Detection Kit (Genecopoeia). Ct values were processed using the 2^−ΔΔCt^ method.

### Nuclear fractionation assay

Circ-ATAD1 subcellular localization in Hs 3.T and MG-63 cells was analyzed using a Nuclear/Cytosol Fractionation Kit (BioVision, # K266). Briefly, cells were fractionated into nuclear and cytoplasm samples and subjected to total RNA extraction followed by RT-PCR to determine circ-ATAD1 expression with GAPDH as the internal control.

### RNA pull-down assay

Premature miR-154-5p and mimic miRNA (no targets in the human genome) were labeled with biotin and named bio-pre-miR-154-5p and bio-mimic miRNA, respectively. Hs 3.T and MG-63 cells were transfected with bio-pre-miR-154-5p or bio-mimic miRNA, and the biotinylated miRNAs were isolated using streptavidin magnetic beads (Invitrogen). Circ-ATAD1 levels in these two pull-down samples were detected using RT-qPCRs.

### Cell invasion and migration assay

The invasion and migration abilities of both Hs 3.T and MG-63 cells were analyzed using Transwell inserts (8 μm, Corning). Prior to invasion assay, insert membranes were coated with Matrigel (Corning) for 8 h. Briefly, cells in non-serum media were added to the upper chamber, and cell invasion and migration were induced for 24 h by media with 20% FBS to the lower chamber. The migrating and invading cells were stained using 0.5% crystal violet (Sigma-Aldrich) and observed and counted under a microscope.

### Statistical analysis

Unpaired t test was used to compare two independent groups, and paired t test was used to compare paired tissues. A *p* < 0.05 was considered statistically significant.

## Results

### Levels of circ-ATAD1 and mature miR-154-5p, but not premature miR-154-5p, were altered in OS

**Fig. 1:**
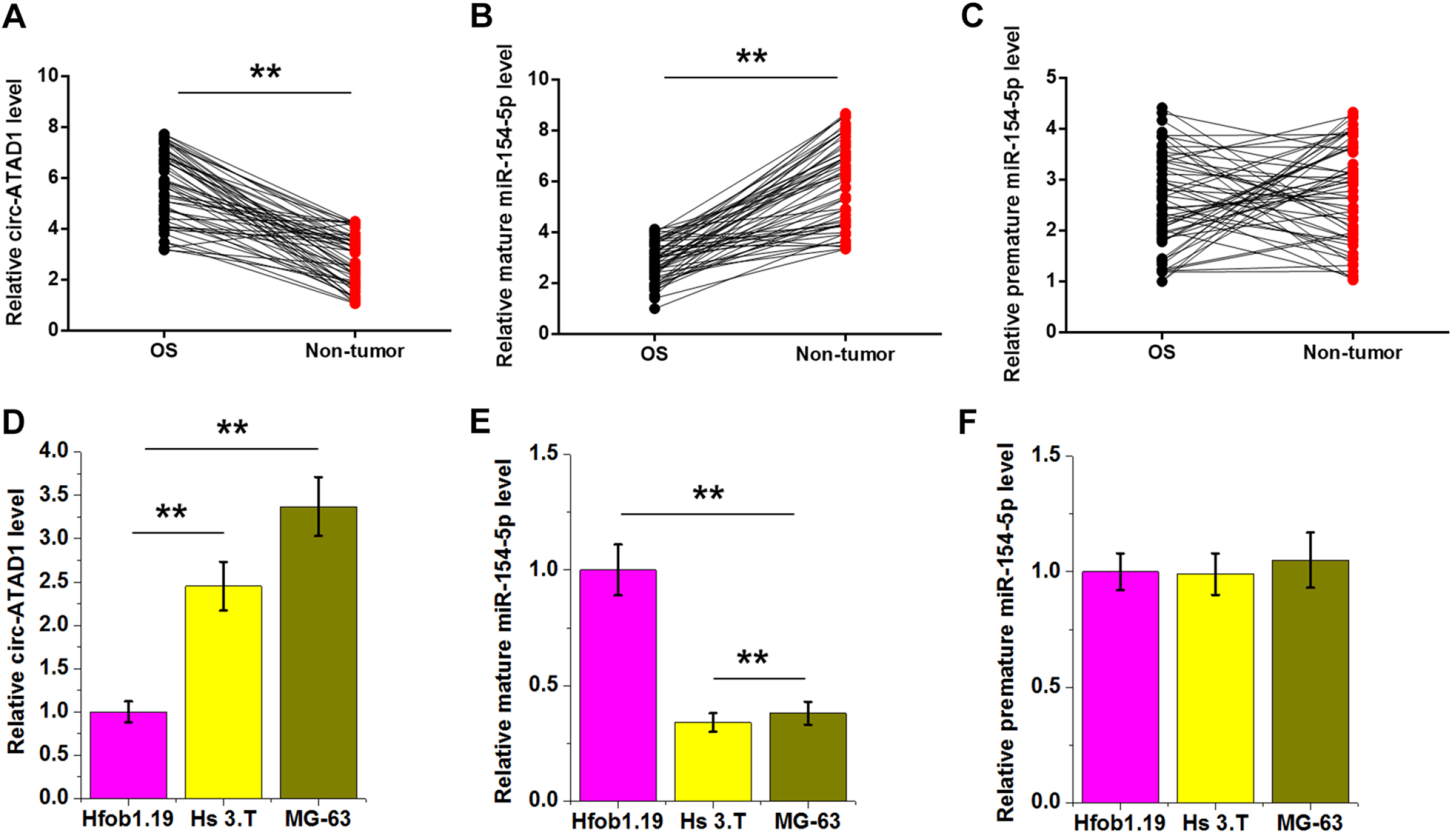
Circ-ATAD1 and mature miR-154-5p, but not premature miR-154-5p, were altered in OS. The levels of circ-ATAD1 (**A**), mature miR-154-5p (**B**), and premature miR-154-5p (**C**) in paired OS and non-tumor tissues from 56 OS patients were determined by RT-qPCR. Gene expression levels were compared by paired t test. Levels of circ-ATAD1 (**D**), mature miR-154-5p (**E**), and premature miR-154-5p (**F**) in Hfob1.19 normal bone cells and OS cell lines Hs 3.T and MG-63 were also analyzed with RT-qPCR. **, *p* < 0.01

The levels of circ-ATAD1, mature miR-154-5p, and premature miR-154-5p in paired OS and non-tumor tissues from 56 OS patients were determined by RT-qPCR. Circ-ATAD1 is overexpressed (Fig. 1A, *p* < 0.01), while mature miR-154-5p was under-expressed in OS tissues (Fig. 1B, *p* < 0.01) than in non-tumor tissues. In contrast, premature miR-154-5p level was not significantly altered (Fig. 1C). The levels of circ-ATAD1, mature miR-154-5p, and premature miR-154-5p in normal bone cell line Hfob1.19 and OS cell lines Hs 3.T and MG-63 were also analyzed using RT-qPCR. Compared to Hfob1.19 cells, Hs 3.T and MG-63 cells exhibited significantly increased circ-ATAD1 (Fig. 1D, *p* < 0.01) and decreased mature miR-154-5p (Fig. 1E, *p* < 0.01) levels. No significant difference in premature miR-154-5p level was observed among the three cell lines.

### Circ-ATAD1 was inversely correlated with mature miR-154-5p, but not premature miR-154-5p

**Fig. 2:**
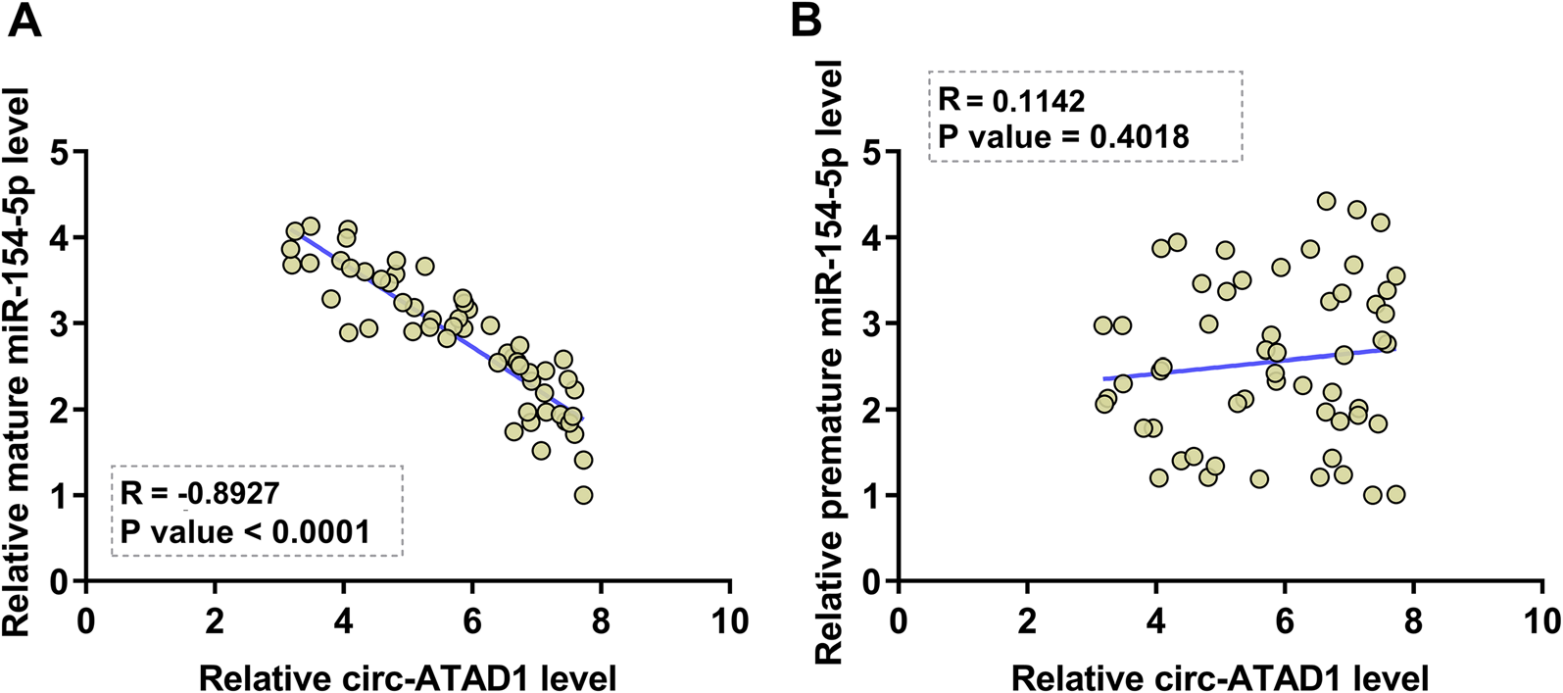
Circ-ATAD1 was inversely correlated with mature miR-154-5p, but not premature miR-154-5p. Pearson correlation coefficient was applied to analyze the correlations between circ-ATAD1 and mature miR-154-5p (**A**) or premature miR-154-5p (**B**) across OS tissues

Pearson’s correlation coefficient analysis showed that circ-ATAD1 levels were inversely and significantly correlated with mature miR-154-5p levels across OS tissues (Fig. 2A). In contrast, circ-ATAD1 and mature miR-154-5p were not significantly correlated with each other (Fig. 2B).

### Circ-ATAD1 is expressed in both the nucleus and cytoplasm and directly interacts with premature miR-154-5p

**Fig. 3:**
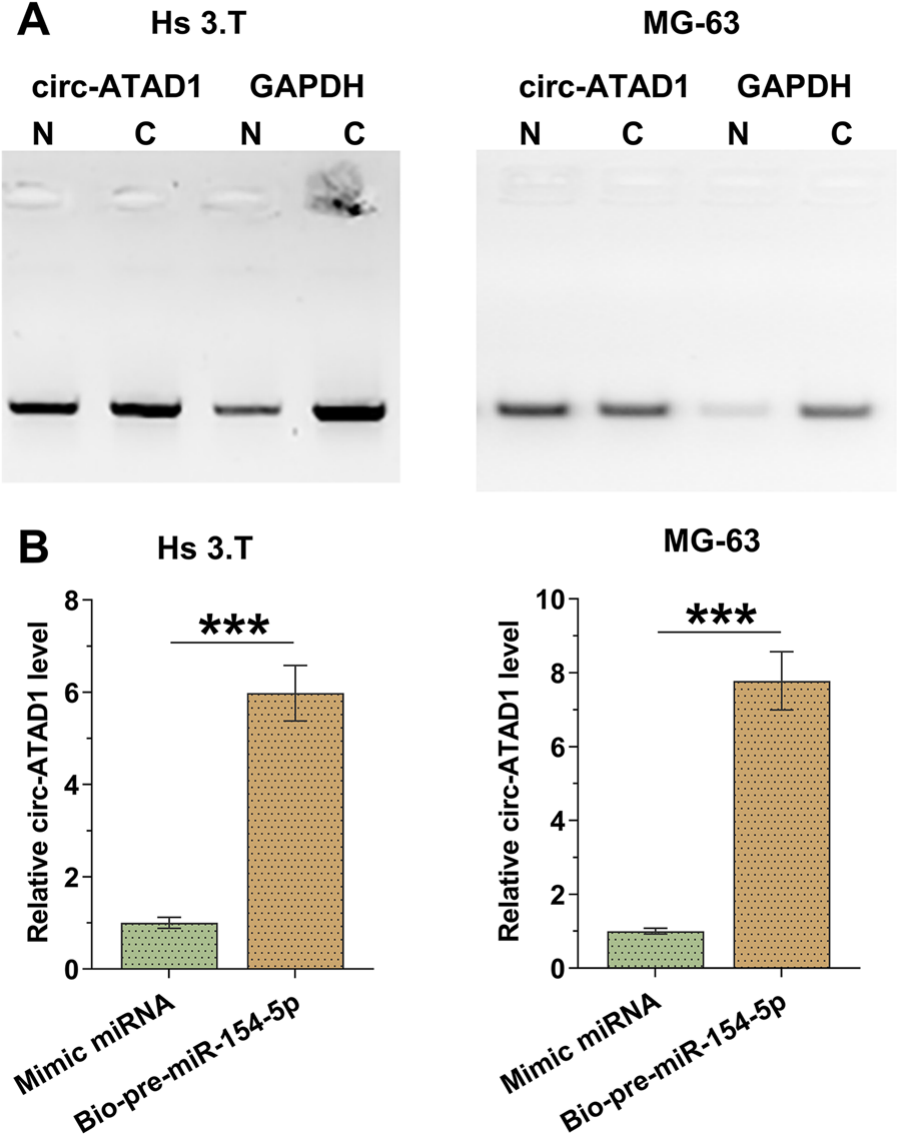
Circ-ATAD1 is expressed in both nuclei and cytoplasm and directly interacts with premature miR-154-5p. The subcellular localization of circ-ATAD1 in Hs 3.T and MG-63 cells was analyzed by nuclear fractionation assay (**A**). RNA pull-down assay was performed to analyze the interaction between circ-ATAD1 and premature miR-154-5p (**B**). N, nuclear, C, cytoplasm; ***, *p* < 0.001

Nuclear fractionation assay was performed to analyze the subcellular location of circ-ATAD1 in both Hs 3.T and MG-63 cells. It was observed that circ-ATAD1 was expressed in both nuclei and cytoplasm of these cells (Fig. 3A). RNA pull-down assay was performed to analyze the interaction between circ-ATAD1 and premature miR-154-5p. Compared to miRNA mimic group, bio-pre-miR-154-5p group showed significantly higher circ-ATAD1 level (Fig. 3B, *p* < 0.001).

### Circ-ATAD1 overexpression suppressed miR-154-5p maturation in Hs 3.T and MG-63 cells

**Fig. 4:**
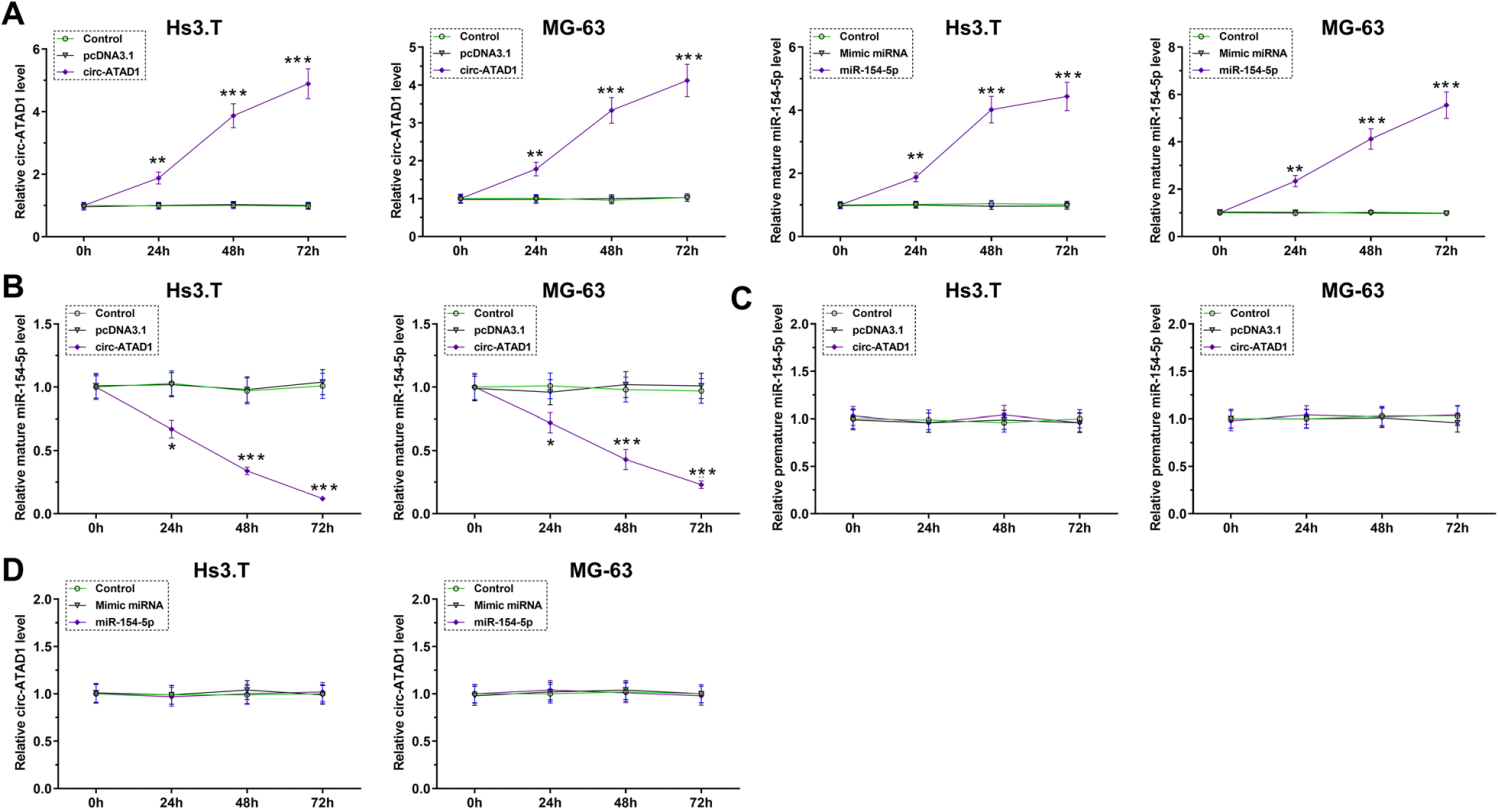
Circ-ATAD1 overexpression suppressed miR-154-5p maturation in Hs 3.T and MG-63 cells. Hs 3.T and MG-63 cells were transfected with circ-ATAD1 expression vector or miR-154-5p mimic, and circ-ATAD1 and miR-154-5p overexpression was confirmed every 24 h until 72 h (**A**). The effects of circ-ATAD1 overexpression on the levels of mature miR-154-5p (**B**) and premature miR-154-5p (**C**) as well as the expression of circ-ATAD1 in cells with miR-154-5p overexpression (**D**) were analyzed by RT-qPCR. *, *p* < 0.05; **, *p* < 0.01; ***, *p* < 0.001

**Fig. 5:**
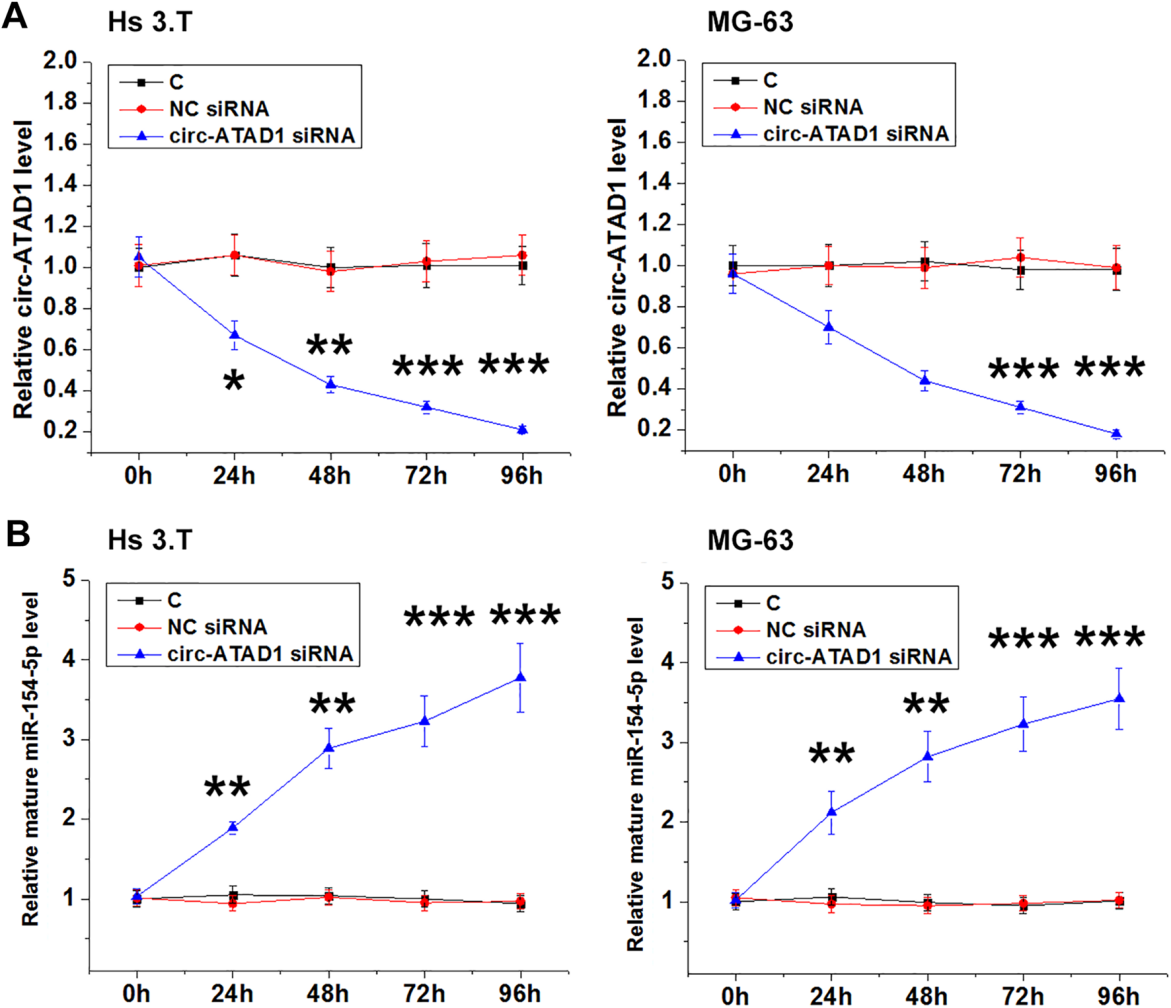
Circ-ATAD1 overexpression increased miR-154-5p level in Hs 3.T and MG-63 cells. SiRNA silencing of circ-ATAD1 was achieved from 24 to 96 h in Hs 3.T and MG-63 cells (**A**). Circ-ATAD1 silencing increased mature miR-154-5p level (**B**). *, *p* < 0.05; **, *p* < 0.01; ***, *p* < 0.001

Hs 3.T and MG-63 cells were transfected with circ-ATAD1 expression vector or miR-154-5p mimic. It was observed that circ-ATAD1 and miR-154-5p were significantly overexpressed between 24 and 72 h compared to control (C) and NC (pcDNA3.1 or NC miRNA) groups (Fig. 4A, *p* < 0.01 or *p* < 0.001). Moreover, circ-ATAD1 overexpression decreased mature miR-154-5p level (Fig. 4B, *p* < 0.05, or *p* < 0.001), but not premature miR-154-5p level (Fig. 4C). Moreover, no significant changes in circ-ATAD1 expression level were observed in cells with miR-154-5p overexpression at each time point (Fig. 4D). SiRNA silencing of circ-ATAD1 was also achieved from 24 to 96 h (Fig. 5A, *p* < 0.05, *p* < 0.01 or *p* < 0.001). It was observed that circ-ATAD1 silencing increased mature miR-154-5p level (Fig. 5B, *p* < 0.01 or *p* < 0.001). PIWIL1 is a confirmed target of miR-154-5p [14]. It was observed that circ-ATAD1 overexpression increased PIWIL1 mRNA level (Additional file 1: Fig. S1, *p* < 0.01 or *p* < 0.001).

### Circ-ATAD1 increased the invasion and migration of both Hs 3.T and MG-63 cells via miR-154-5p

**Fig. 6:**
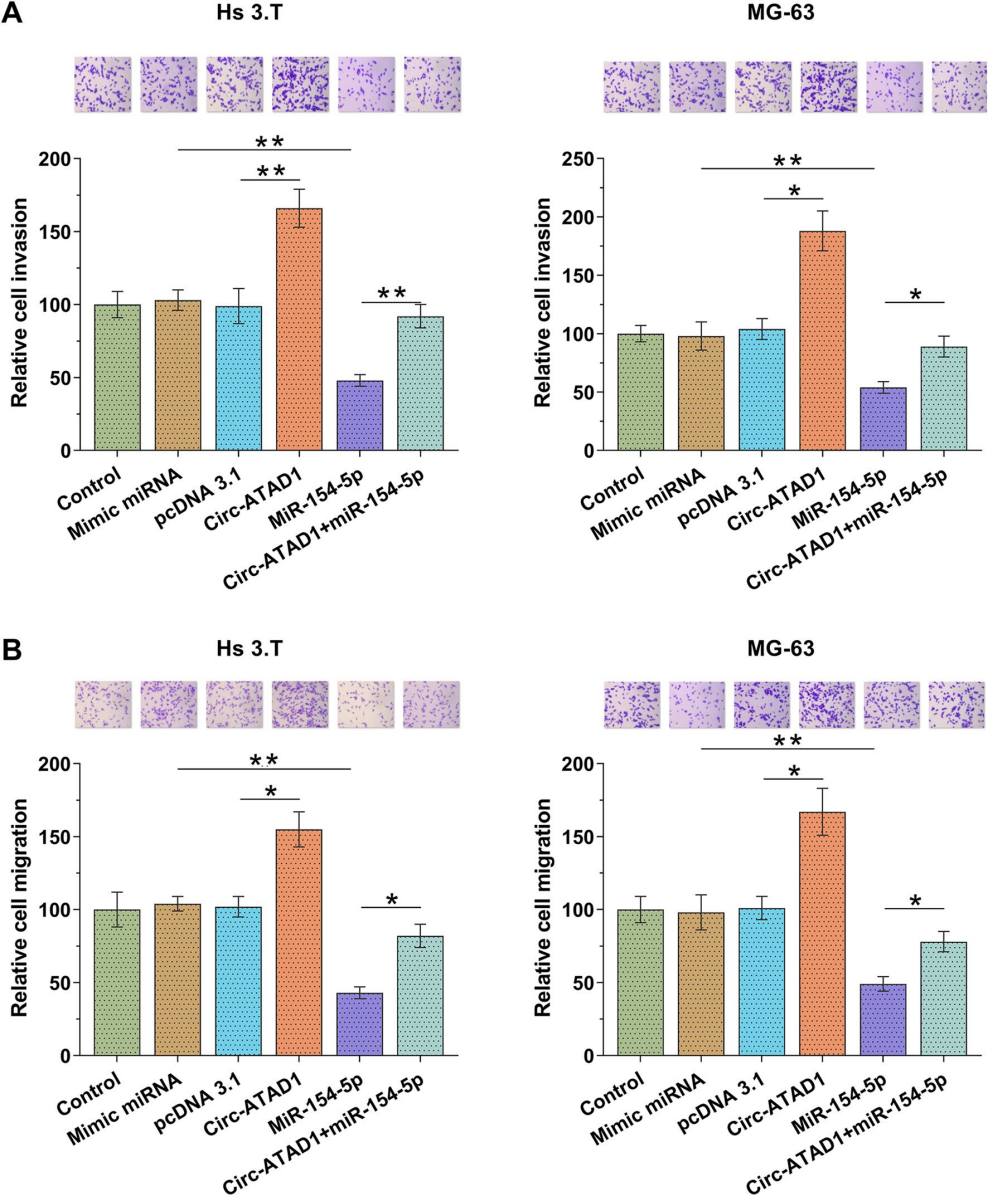
Circ-ATAD1 increased the invasion and migration of both Hs 3.T and MG-63 cells via miR-154-5p. Transwell assays were performed to analyze the roles of circ-ATAD1 and miR-154-5p in regulating OS cell invasion (**A**) and migration (**B**). *, *p* < 0.05; **, *p* < 0.01

Transwell assays were performed to analyze the roles of circ-ATAD1 and miR-154-5p in regulating OS cell invasion and migration. Our data illustrated that circ-ATAD1 overexpression increased cell invasion (Fig. 6A, *p* < 0.05 or *p* < 0.01) and migration (Fig. 6B, *p* < 0.05 or *p* < 0.01) compared to C and NC groups, and mature miR-154-5p overexpression suppressed cell behaviors. In addition, circ-ATAD1 overexpression reduced the effects of mature miR-154-5p overexpression on cell behaviors.

## Discussion

This study explored the interaction between circ-ATAD1 and miR-154-5p in OS and showed that circ-ATAD1 was highly expressed in OS and might inhibit miR-154-5p maturation to attenuate its tumor-suppressive role in OS.

Zhang et al. recently characterized a novel oncogenic circRNA named circ-ATAD1 in gastric cancer [13] and demonstrated that circ-ATAD1 was highly expressed in gastric cancer. In addition, circ-ATAD1 might sponge miR-140-3p to increase YY1 expression. YY1 then binds to PCIF1 promoter to upregulate PCIF1 and promote gastric cancer progression [13]. Based on our knowledge, the function of circ-ATAD1 in other cancers is unknown. In this study, we observed circ-ATAD1 upregulation in OS. In addition, circ-ATAD1 overexpression increased the invasion and migration of two GC cell lines. Therefore, circ-ATAD1 is likely an oncogenic circRNA in GC.

MiR-154-5p plays different roles in different cancers [14–16]. For instance, miR-154-5p is highly expressed in renal cell carcinoma and plays its oncogenic roles by inhibiting cell apoptosis and increasing cell proliferation [15]. In contrast, miR-154-5p was under-expressed in glioblastoma. MiR-154-5p overexpression targets PIWIL1 to suppress cell metastasis and proliferation, suggesting it plays a tumor-suppressive role [16]. Tian et al. reported miR-154-5p downregulation in OS [14]. In addition, miR-154-5p overexpression suppresses OS progression by upregulating Bax and cleaved caspase 3 and downregulating Bcl-2 [14]. Our study confirmed miR-154-5p downregulation in OS and further showed the inhibitory effects of miR-154-5p on OS cell invasion and migration.

Although miR-154-5p has been proven to play critical roles in different cancers [14–16], its upstream regulators in these cancers are unknown. In this study, we showed that circ-ATAD1 could suppress miR-154-5p maturation in OS cells. We also showed that circ-ATAD1 could be detected in both nuclei and cytoplasm. In addition, circ-ATAD1 could directly interact with premature miR-154-5p. Considering the fact that the movement of premature miRNAs from the nucleus to the cytoplasm is essential for miRNA maturation [17], we speculated circ-ATAD1 might suppress the transportation of premature miR-154-5p by directly suppressing its maturation.

Although numerous studies have been performed to explore the roles of ncRNAs in human diseases [18–21], more efforts should be made to further analyze their potentials in disease treatment.

## Conclusion

Circ-ATAD1 is overexpressed in OS and might sponge miR-154-5p to suppress OS cell invasion and migration.

## Supplementary Information

**Additional file 1. Figure S1**. Circ-ATAD1 overexpression increased PIWIL1 mRNA levelThe role of circ-ATAD1 in the expression of PIWIL1, a confirmed target of miR-154-5p, was analyzed with RT-qPCR. **, p<0.01; ***, p<0.001.

## Data Availability

The data are not publicly available due to their containing information that could compromise the privacy of research participants, but are available on reasonable request from the corresponding author.
