# Supplementary figures and images for: Circ-ATAD1 is overexpressed in osteosarcoma (OS) and suppresses the maturation of miR-154-5p to increase cell invasion and migration
Source: J Orthop Surg Res. 2021 Dec 2;16:699. doi: 10.1186/s13018-021-02809-4 (PMC8638470; doi:10.1186/s13018-021-02809-4)

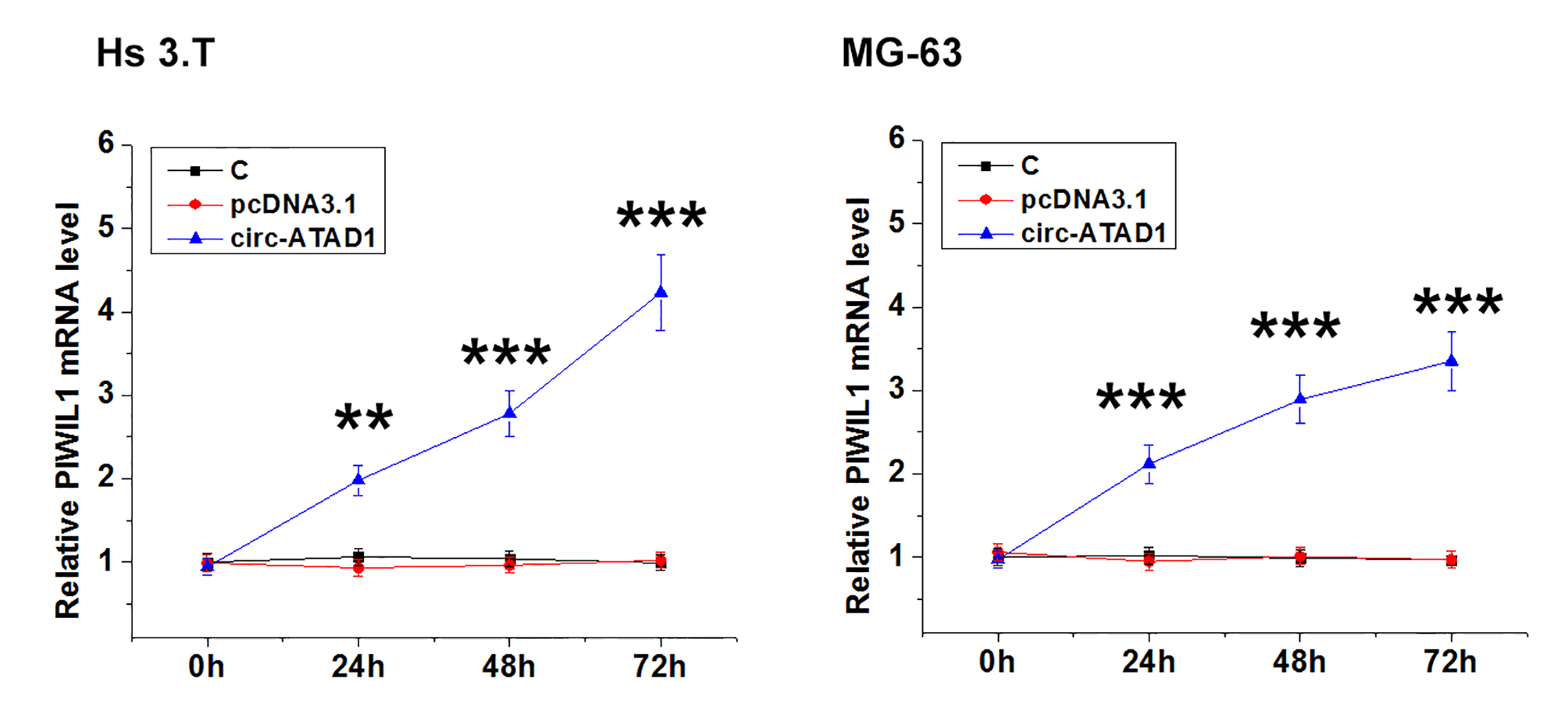

Supplement: Supplementary file 1 — Additional file 1. Figure S1. Circ-ATAD1 overexpression increased PIWIL1 mRNA levelThe role of circ-ATAD1 in the expression of PIWIL1, a confirmed target of miR-154-5p, was analyzed with RT-qPCR. **, p<0.01; ***, p<0.001. [file 13018_2021_2809_MOESM1_ESM.tif]
